# The Perils of Molecular Interpretations from Vibrational Spectra of Complex Samples

**DOI:** 10.1002/anie.202411596

**Published:** 2024-11-07

**Authors:** Tarek Eissa, Liudmila Voronina, Marinus Huber, Frank Fleischmann, Mihaela Žigman

**Affiliations:** ^1^ Ludwig-Maximilians-Universität München (LMU), Chair of Exper-imental Physics - Laser Physics Garching Germany; ^2^ Max Planck Institute of Quantum Optics (MPQ) Laboratory for Attosecond Physics Garching Germany; ^3^ Technical University of Munich (TUM), School of Computation, Information and Technology Garching Germany

**Keywords:** Vibrational Spectroscopy, Infrared Spectroscopy, Molecular Interpretation, Spectral Assignment, Blood Analysis

## Abstract

Vibrational spectroscopy is a widely used technique for chemical characterizations across various analytical sciences. Its applications are increasingly extending to the analysis of complex samples such as biofluids, providing high‐throughput molecular profiling. While powerful, the technique suffers from an inherent limitation: The overlap of absorption information across different spectral domains hinders the capacity to identify individual molecular substances contributing to measured signals. Despite the awareness of this challenge, the difficulty of analyzing multi‐molecular spectra is often underestimated, leading to unsubstantiated molecular interpretations. Here, we examine the prevalent overreliance on spectral band assignment and illuminate the pitfalls of correlating spectral signals to discrete molecular entities or physiological states without rigorous validation. Focusing on blood‐based infrared spectroscopy, we provide examples illustrating how peak overlap among different substances, relative substance concentrations, and preprocessing steps can lead to erroneous interpretations. We advocate for a viewpoint shift towards a more careful understanding of complex spectra, which shall lead to either accepting their fingerprinting nature and leveraging machine learning analysis – or involving additional measurement modalities for robust molecular interpretations. Aiming to help translate and improve analytical practices within the field, we highlight the limitations of molecular interpretations and feature their viable applications.

## Graphical abstract

Vibrational spectroscopy is a powerful molecular fingerprinting tool. Yet, interpreting IR spectra of molecularly complex media is challenging. Here, we examine the often‐overlooked intricacies of spectral data analyses, revealing commonly misleading interpretations. Focusing on blood‐based IR spectroscopy, we argue for a viewpoint shift to accept the technique's fingerprinting nature and provide suggestions to improve analytical practices.

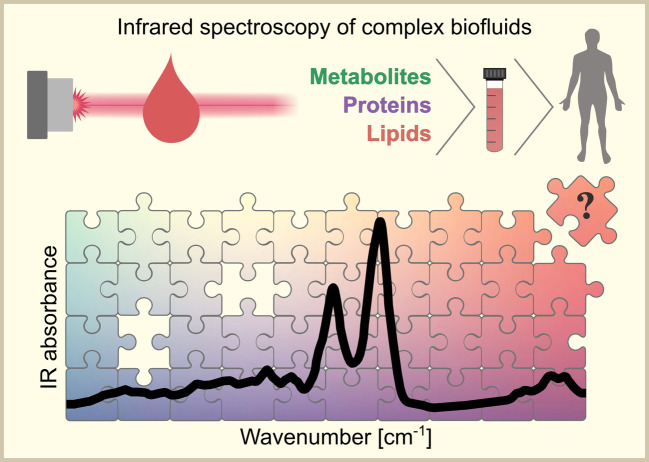

## Introduction

Infrared (IR) spectroscopy has traditionally been taught as a powerful modality for analyzing the structure of individual organic compounds, identifying simple chemicals, or for use in industrial quality control and process monitoring procedures.[^1^, ^2^, ^3^, ^4^, ^5^] More recently, its applications have expanded to the quantitative analysis of complex multi‐molecular mixtures, including innovative applications in biomedical spectroscopy and photonics.[^1^, ^2^, ^6^, ^7^, ^8^, ^9^, ^10^, ^11^, ^12^, ^13^, ^14^] This expansion is partly attributed to advancements in instrumentation – such as improved spectral resolution, a broader availability of Fourier transform IR (FTIR) spectrometers, and, lately, laser‐based spectroscopic methods.[^1^, ^2^, ^15^, ^16^, ^17^, ^18^, ^19^] Additionally, progress in computational methods and molecular biology have further driven IR spectroscopy to become a prominent tool for high‐throughput probing of biological specimens.[^1^, ^2^, ^8^, ^20^]

IR spectroscopy probes the composition of a given mixture by simultaneously measuring the resonant vibrational response of present molecular structures when excited by IR radiation. The principal advantage of the method is its ability to deliver information on a wide variety of molecular constituents within a sample in a label‐free one‐shot measurement, requiring no prior knowledge and minimal sample preparation. When analyzing single substances or mixtures of few organic molecules, it is generally possible to distinguish between spectroscopic signatures of different molecular functional groups, rely on well‐defined spectral band patterns, and thus identify specific substances with high confidence.[^1^, ^3^, ^4^] The former cannot be performed when analyzing spectra of more complex multi‐molecular samples, such as biofluids, cellular components, or other biological media. The overlap in absorption peaks or interference between different molecular components hinders the capacity to link spectral signals of different functional groups to specific substances or even molecular classes (e.g., carbohydrates, lipids, or proteins). Thus, while IR spectroscopy is highly specific to identifying functional groups, performing peak interpretations can be challenging, and the method generally suffers from low molecular specificity when analyzing complex mixtures to profile biological systems.[^21^, ^22^, ^23^, ^24^, ^25^]

Nevertheless, the desire to link spectral signals to specific substances in a clinical scenario is omnipresent, and misinterpretations of complex spectra remain widespread across peer‐reviewed literature. In our experience, the question about the underlying molecular origin of spectral signals often arises in the scientific discourse. This is usually not the right question or viewpoint, as its answers typically depend on oversimplified assumptions. This situation underscores a broader issue: The classical teaching of IR spectroscopy, which serves well for simple matrices, does not directly extend to the analysis of molecularly complex biological media. While many experts in the field are aware and share this same perspective, there is still a pressing need for a broader viewpoint shift in how spectral data is interpreted and presented, moving towards a framework that accommodates the complexity of biological systems.

In this Scientific Perspective, we critically examine the limitations inherent to analyzing complex spectra – a topic that, in our opinion, needs to be more directly and comprehensively addressed in scientific literature. We focus on IR spectroscopy of human blood derivatives – native serum and plasma. We highlight examples from previous studies where molecular interpretations range from reaching reasonable and most likely correct conclusions to ones that are highly speculative and questionable. Using data from several case‐control and health diagnostics scenarios we have considered previously, we demonstrate (i) how molecular interpretations can be misled by the overlap of spectrally similar yet biologically very different molecular substances; (ii) how measurement preprocessing can affect data interpretations; and (iii) how linking low‐abundant substances, e.g., DNA/RNA, or protein biomarkers such as cancer antigen 125 (CA125) or prostate‐specific antigen (PSA), to spectral signals is compromised by measurement sensitivity and background variability. Our analyses and conclusions are based on different case examples of molecules found in cell‐free human blood – proteins, lipid particles, nucleic acids, and water‐soluble metabolites (Supporting Information Table S1) – for which we measured IR spectra. We further utilize spectra from several thousand blood serum and plasma samples experimentally acquired in our laboratory over the last decade.

Ultimately, we urge users and professionals in the field of biomedical IR spectroscopy to accept the approach's fingerprinting nature, which inherently lacks sufficient molecular specificity. Importantly, this property does not prevent IR spectra from being specific to an individual or a physiological state. To fully capture the informational content of IR molecular fingerprints, appropriate machine learning approaches can be used.[^1^, ^7^, ^24^, ^26^, ^27^, ^28^] In contexts where molecular interpretation of experimental IR spectroscopic signatures is deemed necessary, orthogonal molecular‐specific approaches should be involved, or the sample should be prepared in a way that reduces its molecular complexity.[^10^, ^29^, ^30^, ^31^, ^32^, ^33^] While we focus on IR spectroscopy of liquid blood derivatives, the principles we discuss generally apply to vibrational spectroscopy of diverse biological samples (e.g., cerebrospinal fluid or interstitial fluid).^[34]^ We aim to help and guide the establishment of vibrational spectroscopy as a reliable biomedical tool while acknowledging its limitations. We believe that the proposed approach shall foster broader acceptance of the technological strengths inherent to the technique and support bridging the gap to bring the technology closer to clinical applications.

### Misleading Assignments of Spectral Regions to Molecular Classes

**Figure 1:**
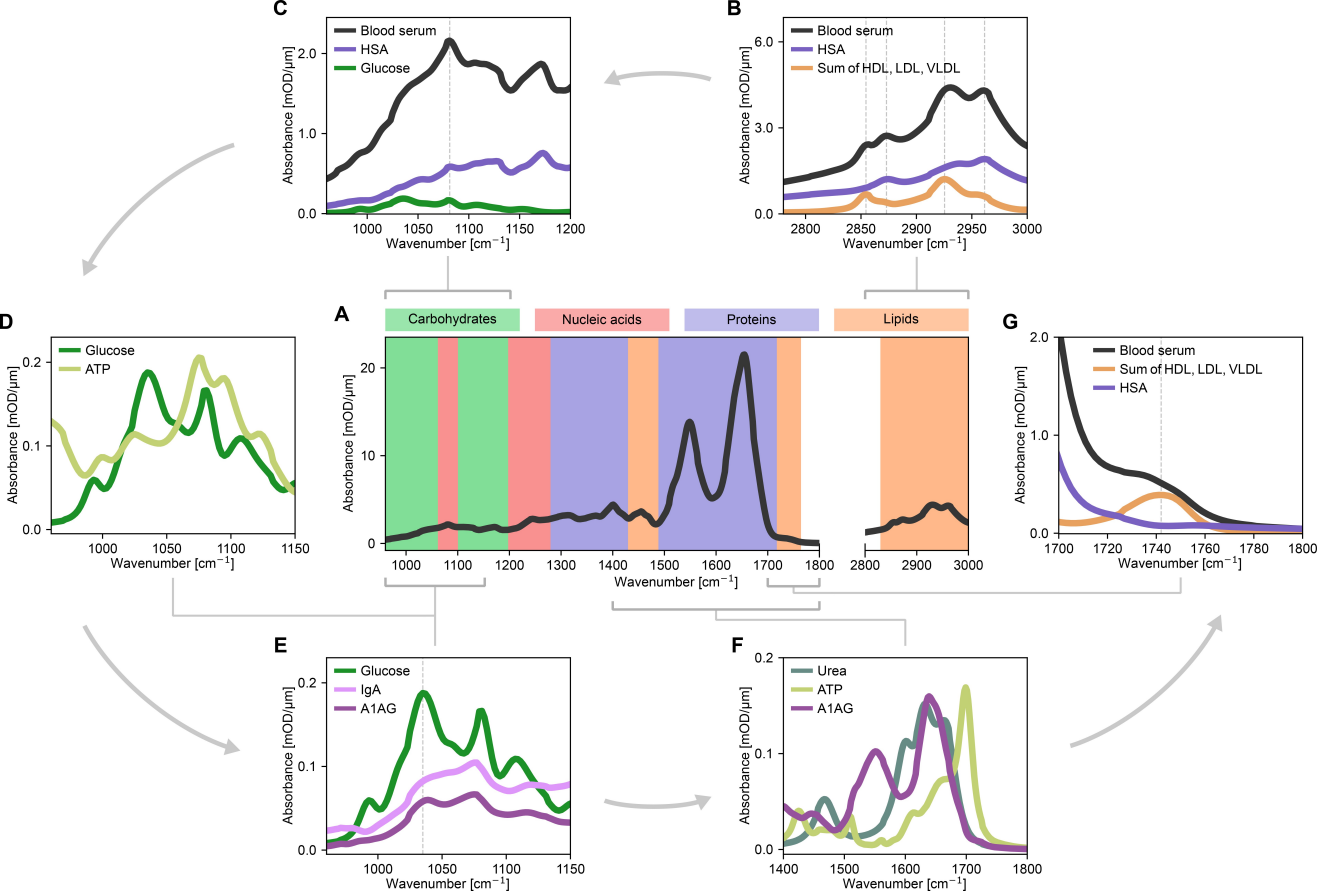
Misleading molecular assignments of spectral regions. (**A**) Descriptive labeling of the mid‐IR commonly assumed in scientific literature to link different molecular classes to discrete spectral regions. A typical spectrum of human blood serum is shown in black. (**B**‐**G**) Comparison between the absorption of various blood serum components recorded separately and scaled by their typical concentrations in healthy individuals (Supporting Information Table S1). Gray arrows indicate the counterclockwise sequence of the panels. *HSA ‐ Human serum albumin; HDL ‐ High‐density lipoprotein; LDL ‐ Low‐density lipoprotein; VLDL ‐ Very‐low‐density lipoprotein; ATP ‐ Adenosine triphosphate; IgA ‐ Immunoglobulin A; A1AG ‐ Alpha‐1‐acid glycoprotein*.

The desire to systematically interpret the information encoded in complex spectra has led to categorizing the mid‐IR into distinct regions associated with specific molecular classes. This resulted in the emergence of depictions within scientific literature that typically define discrete spectral regions responsible for protein, lipid, carbohydrate, and nucleic acid absorption.[^35^, ^36^, ^37^, ^38^, ^39^, ^40^] We provide one example of such a representation in Figure 1A. While the majority of absorption signals of these molecular classes are indeed present within the defined spectral regions, several important issues render this approach oversimplified and can encourage a perspective that overlooks complexities in peak interpretations.

First, there is the straightforward yet important consideration of the relative concentrations of different molecular classes in cell‐free human venous blood. The most prominent absorption bands of any single protein are the amide‐I and ‐II bands, located between 1500–1700 cm^–1^.[^2^, ^41^] Proteins, however, have other weaker absorption bands that cover all the “fingerprint” region that spans 960–1500 cm^–1^, as well as the 2800–3000 cm^–1^ range.^[2]^ On the other hand, the IR absorption of glucose – the most abundant dissolved carbohydrate in blood plasma – is indeed strongest in the region 960–1200 cm^–1^, and the absorption bands of lipids do appear in the regions labeled in Figure 1A. The total absorption of any molecular class, however, is a sum of the products of concentrations of each constituent and their absorption spectrum. The high concentrations of many proteins make their absorption the strongest in any of the regions mentioned above. To demonstrate this, we compare the absorption of the single most abundant protein in cell‐free blood – human serum albumin (HSA) – to the absorption of lipid particles (Figure 1B) and glucose (Figure 1C). Evidently, the absorption of HSA alone is at least as significant in these regions as any of the other molecules in the so‐called “lipid” and “carbohydrate” regions, or even more dominant.

The considerations mentioned above are not limited to the abundance of proteins. For instance, some water‐soluble metabolites contribute to the absorption between 960–1150 cm^–1^, making the “carbohydrate” label of this spectral region also questionable. As an example, we show that the IR absorption of glucose in its typical concentration is comparable to that of adenosine‐triphosphate (ATP), a molecule that does not belong to the carbohydrate class (Figure 1D). As for the “nucleic acid” region highlighted in Figure 1A, it should be emphasized that the IR absorption of DNA and RNA — the primary nucleic acids – cannot be attributed to any vibrational response of cell‐free blood. These substances are present in concentrations far below the detection limits of the current technology – a point that we further address and discuss later in the text.

Importantly, dividing the spectral regions into the four categories (proteins, carbohydrates, lipids, and nucleotides) does not reflect the actual composition of cell‐free blood. Lipids, for instance, are not water‐soluble and are present in blood plasma as part of lipoprotein particles – mostly HDL, LDL, and VLDL – in which triacylglycerols, cholesterol, and phospholipids are found in various proportions.[^42^, ^43^] Furthermore, lipid particles contain a high percentage of proteins, up to 33 % in the case of HDL.^[43]^ As a result, the experimental IR absorption spectra of HDL, LDL, and VLDL contain a clear protein spectral signature, in addition to the absorption bands in the lipid region (Supplementary Figure S1). Thus, while strong lipid absorption signals can appear within the highlighted regions shown in Figure 1A, their absorbance should not be confined to such a restricted range, as an ensemble of diverse molecules compose each lipid particle.

Carbohydrates are primarily present in cell‐free blood not as separate molecules but as glycans – bound to proteins in the process of their post‐translational modification.^[44]^ Due to the chemical similarity of free carbohydrates (e.g., glucose) and glycans, attributing a spectral change to an alteration in the former or the latter is challenging without additional analysis. To illustrate this, we compare the absorption of two glycosylated proteins in their typical concentrations to that of glucose (Figure 1E). It is evident that the most prominent absorption peak of glucose overlaps with the spectral band present in the glycoprotein vibrational spectra.

The examples discussed so far may suggest that one can confidently attribute spectral contributions of proteins, given their high concentrations. We challenge this assumption in Figure 1F, which shows the amide‐I spectral region that is typically used to describe changes in the protein secondary structure.[^2^, ^22^, ^41^, ^45^, ^46^, ^47^, ^48^, ^49^] We depict the IR absorption of alpha‐1‐acid glycoprotein (A1AG), one of the twelve most abundant proteins in blood plasma, compared to the absorption of urea and ATP. It is evident that both metabolites exhibit absorption peaks that could be mistaken for a change in the protein's secondary structure. This purely spectroscopic consideration leads to more general biomedical concerns about using protein secondary structure change as a biomarker, as discussed previously.[^21^, ^22^]

It is also important to acknowledge that there exists a spectral region that can be quite confidently correlated with a specific molecular class – the peak centered around 1742 cm^–1^ (Figure 1G). The total absorbance in this region is predominantly due to the presence of lipid particles. Therefore, it would be reasonable to infer a lipid‐centric contribution when a strong signal is observed there, while considering the overall molecular composition of lipid particles as discussed above.

Given these considerations, we advocate for caution when using descriptive labels for discrete regions of the IR spectrum, even when only intended as a foundational guide. Such labels may lead researchers to misinterpret the spectral signals they observe, which often requires a more critical perspective and experimentation with additional molecular techniques.

### Spectral Similarity of Different Substances

**Figure 2:**
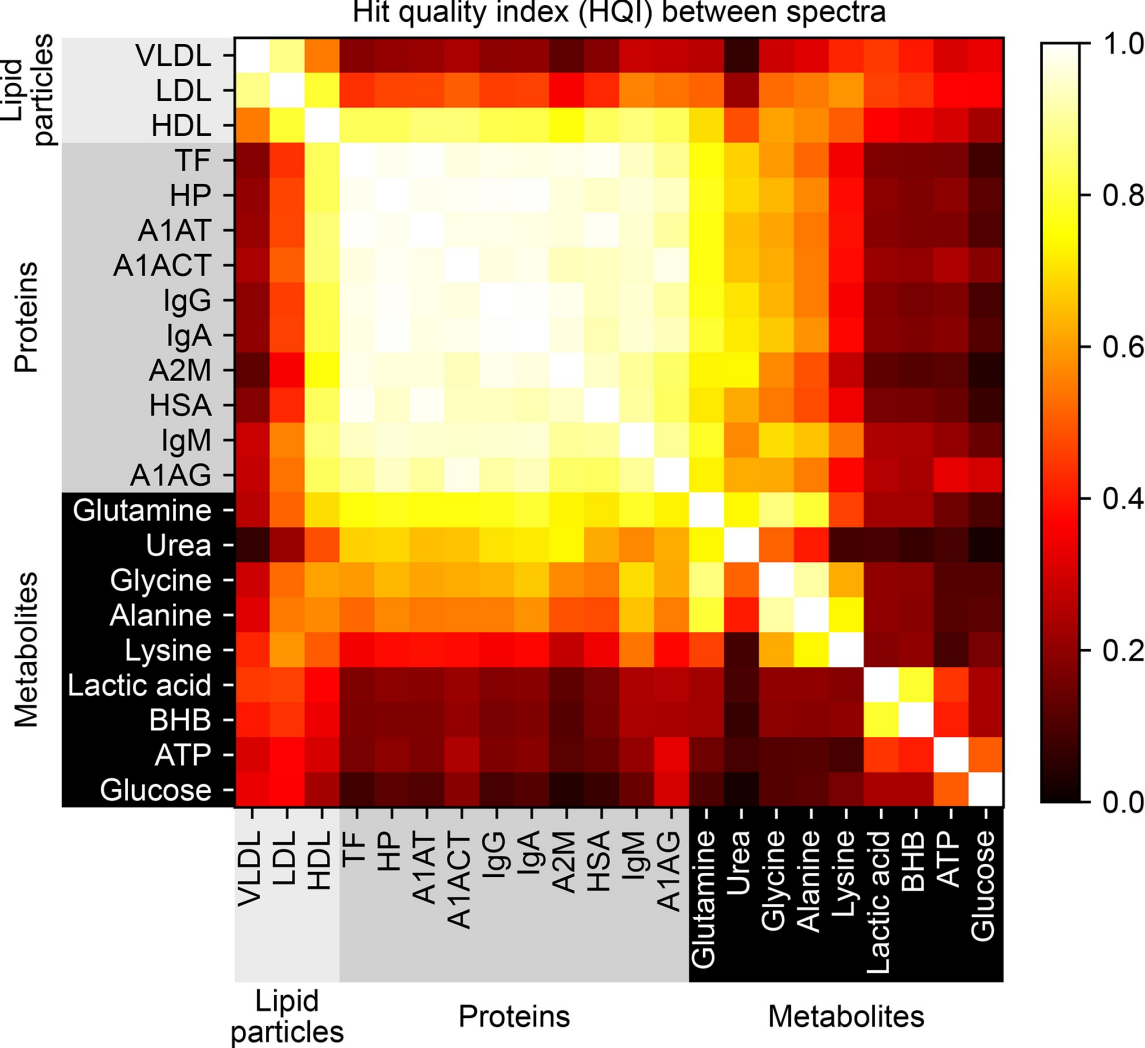
Similarity among IR spectra of organic substances present in blood. Color intensity represents the level of similarity between pairs of IR absorption spectra (960–3000 cm^−^) of single substances. Brighter colors indicate higher spectral overlap. *VLDL ‐ Very low‐density lipoprotein; LDL ‐ Low‐density lipoprotein; HDL ‐ High‐density lipoprotein; TF ‐ Transferrin; HP ‐ Haptoglobin; A1AT ‐ Alpha‐1‐antitrypsin; A1ACT ‐ Alpha‐1‐antichymotrypsin; IgG ‐ Immunoglobulin G; IgA ‐ Immunoglobulin A; A2 M ‐ Alpha‐2‐macroglobulin; HSA ‐ Human serum albumin; IgM ‐ Immunoglobulin M; A1AG ‐ Alpha‐1‐acid glycoprotein; BHB ‐ Beta‐hydroxybutyric acid; ATP ‐ Adenosine triphosphate*.

Having demonstrated the limitations of attributing spectral regions to molecular classes, we now take a more quantitative approach to illustrate the potential for overlap among the spectra of individual organic substances (Figure 2). We calculated the hit quality index (HQI)^[50]^ between pairs of various substances to assess the IR absorption similarity of different components found in cell‐free blood. These include the spectra of lipid particles, proteins, and water‐soluble metabolites. Given two spectra, represented as one‐dimensional vectors u and v of absorption values, the HQI measures the squared cosine of the angle between the two vectors [Eq. 1].

(1) $$HQI=\frac{{\left(u\cdot v\right)}^{2}}{\left(u\cdot u\right)\left(v\cdot v\right)}$$

HQI values closer to one indicate higher collinearity and, therefore, more similar spectral profiles.

The spectra of all investigated individual proteins were found to be very similar to one another (Figure 2). This stands to reason, as proteins have a large molecular structure made of amino acids that share similar functional groups (namely, the amide bonds). Yet, this analysis clearly demonstrates that linking any spectral signal to a single protein “biomarker” is likely to be flawed, as variations in the concentration of nearly any protein could produce comparable spectral signals.

Lipid particles are very complex, comprising a mix of cholesterol, triglycerides, phospholipids, and proteins. This leads to a broad range of vibrational modes. Notably, the spectra of LDL and VLDL showed considerable similarity to one another (Figure 2, see also Figure S1). However, HDL, being enriched with apolipoprotein A1, reflected a spectral profile more closely aligned with that of single proteins than either LDL or VLDL did.

Comparatively, metabolites are smaller and more diverse in terms of their chemical structures and functional groups. This diversity leads to more unique vibrational modes for each metabolite and narrow peaks, resulting in more distinct IR spectra (Figure 2). Nevertheless, some metabolite spectra share similarities to protein spectra. For example, urea – as we previously mentioned, and was highlighted by Ollesch et al.^[51]^ – and, naturally, amino acids, such as glutamine, share these similarities. Furthermore, some metabolite pairs, such as glycine and alanine or lactic acid and beta‐hydroxybutyric acid (BHB), can share similar spectral profiles with one another. Therefore, caution should be taken even when linking spectral signals to smaller molecules. Out of all the substances investigated, glucose was found to generally have the most unique spectral profile, which likely explains the high efficacy of quantifying its concentrations from IR spectra of blood plasma, serum, and whole blood.[^14^, ^52^, ^53^, ^54^, ^55^]

### Ambiguity of Signals in Complex Mixtures

**Figure 3:**
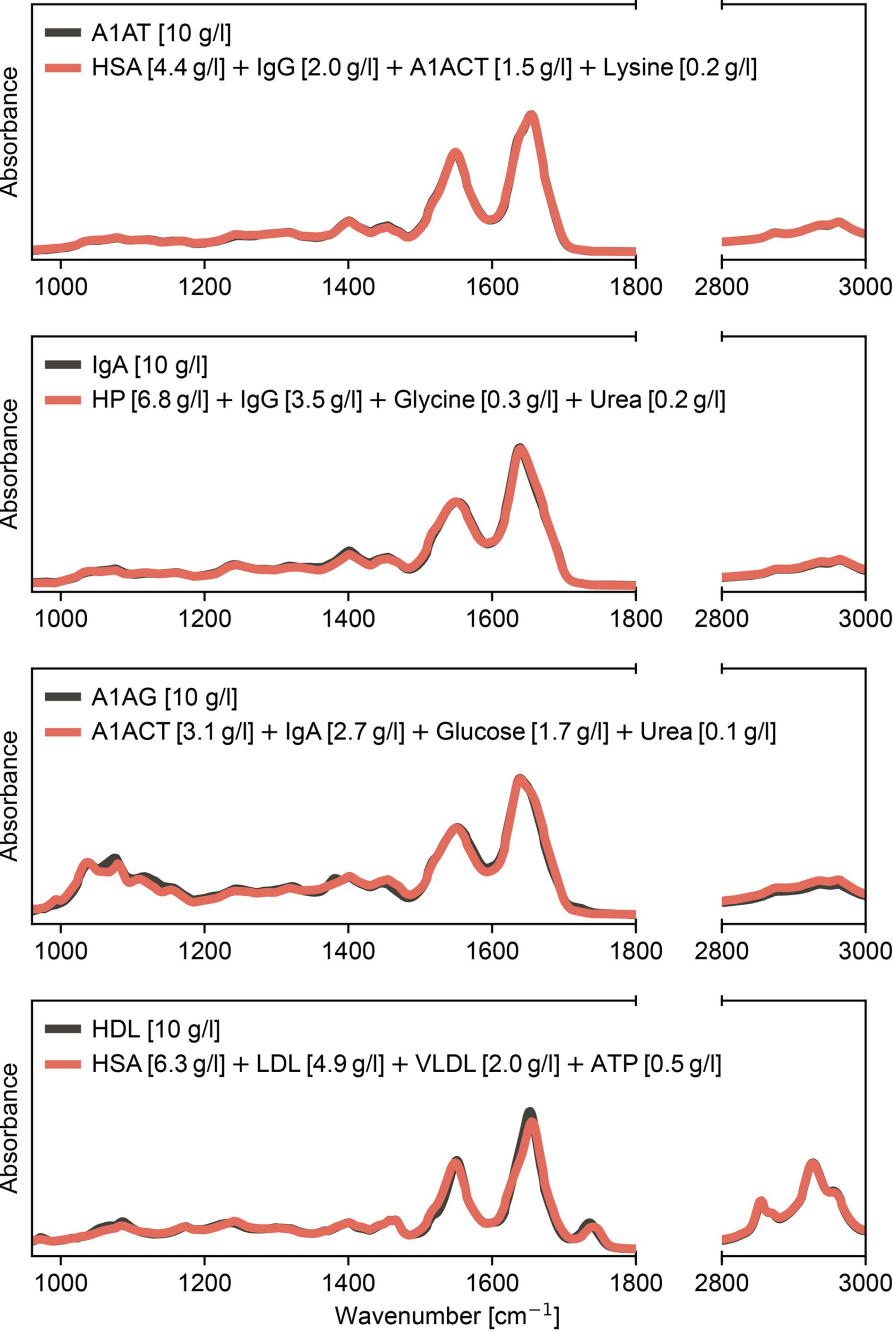
Ambiguity of individual substances in complex mixtures. Spectra of individual substances (black) of larger molecular structures were reconstructed by combining the spectra of other substances (red). The linear combinations were determined by scaling each component with the listed concentrations to yield a reproduced spectrum of the target at 10 g/l. Supplementary Figure S2 depicts the same analysis on an expanded set of proteins and lipid particles. *A1AT ‐ Alpha‐1‐antitrypsin; HSA ‐ Human serum albumin; IgG ‐ Immunoglobulin G; A1ACT ‐ Alpha‐1‐antichymotrypsin; IgA ‐ Immunoglobulin A; HP ‐ Haptoglobin; A1AG ‐ Alpha‐1‐acid glycoprotein; HDL ‐ High‐density lipoprotein; LDL ‐ Low‐density lipoprotein; ATP ‐ Adenosine triphosphate; VLDL ‐ Very‐low‐density lipoprotein*.

While the spectra of individual organic substances can be very similar, the problem of overlapping signals becomes much more prominent when considering the plethora of substances in biological samples. Take a health phenotyping scenario as an example. The typical study reveals how some spectral features significantly differ between two (or more) phenotypes of interest. In many instances, a discussion ensues that relies on peak positions to decipher the bonds and chemical nature of the underlying molecules responsible for such spectral differences.[^45^, ^47^, ^48^, ^56^, ^57^, ^58^, ^59^, ^60^, ^61^, ^62^, ^63^, ^64^] Here, we illustrate how the signal of one substance can be almost perfectly replicated by a combination of only a few other substances (Figure 3) – deeming such speculative assignments unproven.

We modeled the spectra of different proteins and lipid particles as a linear combination of other substances [Eq. 2].

(2) $$y\approx \sum_{i=1}^{m}x_{i}\cdot c_{i}$$

y represents the target spectrum to be reproduced at a given concentration, $x_{i}$ are spectra of other substances, and *c_i_* represents their respective concentrations. The concentrations *c_i_* were determined by a computational optimization that minimizes the reconstruction error. To keep it simple, we limited the number of substances used to reconstruct the target to four (m=4 ).

We found that the IR spectra of all the proteins and lipid particles we considered could indeed be reproduced to a high degree, while only using relatively few substances (Figure 3, Supplementary Figure S2). Interestingly, the metabolite spectra were heavily utilized in reconstructing the target to capture the smaller peaks apparent within the larger molecular structures. This analysis demonstrates, albeit with an extreme example, that observing an IR spectral signal that resembles an individual substance may indeed be caused by a mix of other possibly fully independent molecules that may not even contribute to the same signaling pathways nor be mechanistically interconnected.

**Figure 4:**
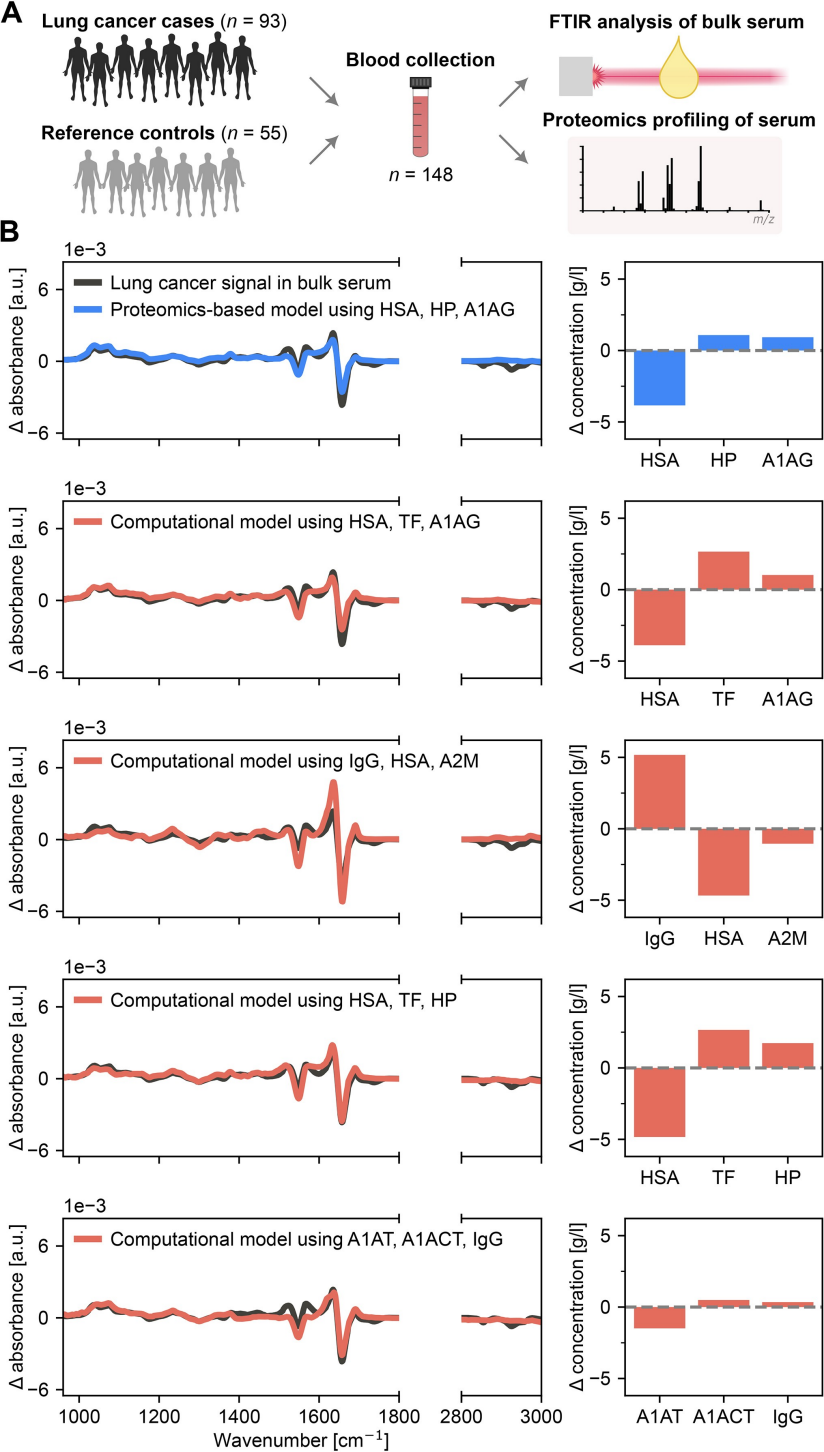
Replicating lung cancer‐induced spectral deviations with different proteins. (**A**) Blood serum was collected from case and reference control individuals to investigate spectral changes due to presence of lung cancer. Two measurements were performed on the same sample sets: Bulk serum IR absorption spectroscopy and proteomics‐based profiling, as described in detail previously.^[29]^ (**B**) Black curves depict the difference in the mean IR measurements between case and control individuals. Blue curve (upper panel) depicts a reproduced signal based on the three proteins that changed the most between case and control individuals, as determined through proteomics. Remaining curves (red) reproduce the same signal with other combinations of three proteins that were determined computationally (i.e., without direct evidence linking this protein combination to the true signal). Bar graphs depict the concentration changes in the modeled proteins that yielded the blue and red curves. *HSA ‐ Human serum albumin; HP ‐ Haptoglobin; A1AG ‐ Alpha‐1‐acid glycoprotein; TF ‐ Transferrin; IgG ‐ Immunoglobulin G; A2 M ‐ Alpha‐2‐macroglobulin; A1AT ‐ Alpha‐1‐antitrypsin; A1ACT ‐ Alpha‐1‐antichymotrypsin; IgG ‐ Immunoglobulin G*.

To provide a parallel demonstration of a more realistic application, we reconsidered one of our previous works.^[29]^ This study investigated a case‐control biomedical diagnostic application to detect spectral differences due to lung cancer with FTIR spectroscopy of blood serum. Mass spectrometry‐based proteomics profiling of the same sample set helped identify the differences in protein concentrations between cases and controls (Figure 4A). In the prior work, we found that the differences between the spectra of case and control individuals observed in bulk serum spectra could be largely reproduced from a reduced molecular model that relied on three proteins (Figure 4B, upper panel). These proteins (HSA, HP, and A1AG) were identified as the ones that changed the most in absolute concentration between the case and control individuals, as measured by proteomics.

Now, we demonstrate that this very previously described lung cancer IR signal can be derived when other combinations of three individual proteins are considered (Figure 4B, remaining panels). Interestingly, in one of the four examples we present, the lung cancer signal could be largely replicated with changes in concentrations of A1AT, A1ACT, and IgG – three proteins that were not even relevant in the proteomics‐based model. This further showcases that without supplementing the IR approach with yet further analytical methods capable of molecular identification (e.g., various ‐omics approaches), conclusions on which molecules contribute to discriminative signals can very likely lead to inaccurate conclusions.

### Signals ofLow Abundant Molecules

**Figure 5:**
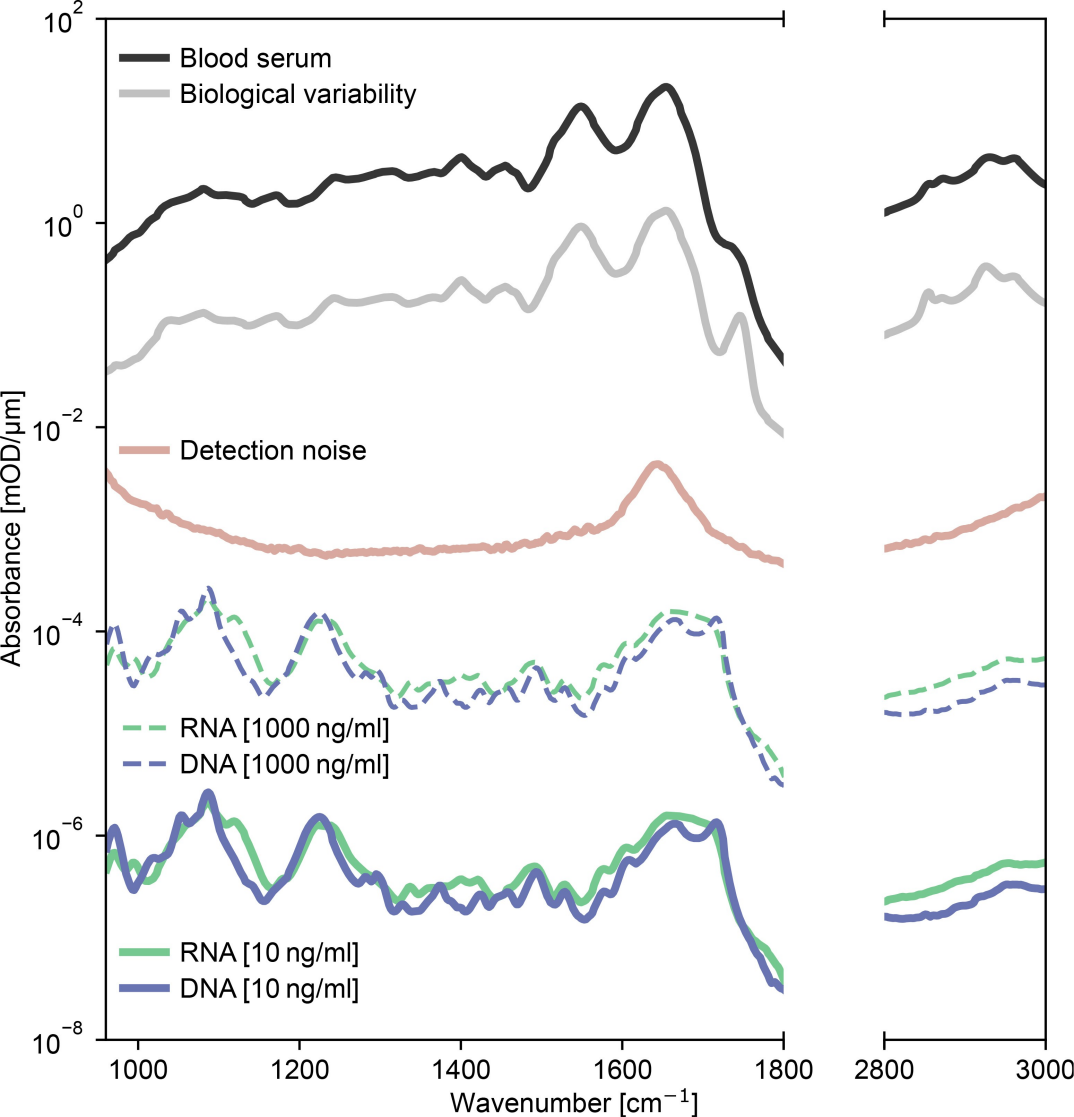
Contributions of cell‐free nucleic acids relative to IR detection limits. Black curve depicts the IR absorbance of a typical human serum sample. Gray curve depicts the standard deviation of serum spectra across different individuals. Brown curve depicts the detection noise of the IR measurement, determined by calculating the standard deviation of repeated measurements of a technical replicate (pure water). Dashed curves depict the IR absorbance spectra of RNA (green) and DNA (blue) at 100x their median concentrations in cell‐free blood, while solid lines represent their absorbance at their typical median concentrations. Y‐axis is plotted on a logarithmic scale.

In some cases apparent in scientific literature, molecules of a very low concentration are linked to specific wavenumbers and suggested to be responsible for observed signals in the analyzed specimens.[^45^, ^46^, ^61^, ^62^, ^64^, ^65^, ^66^, ^67^, ^68^, ^69^, ^70^] Typical examples are circulating cell‐free RNA/DNA and known biomarkers such as CA125, mannose, or PSA. Using cell‐free DNA and RNA as case examples, here we demonstrate that such substances are so far below the detection limit that the measurement variability certainly conceals their expected contribution to the possibly detectable IR spectral signals (Figure 5).

The median concentrations of circulating DNA and RNA have been reported to range between 6.8–10 ng/ml in cell‐free blood.[^71^, ^72^, ^73^] Highest individual concentrations can reach near 100 ng/ml. At their median concentrations, these substances are around three orders of magnitude below the detection limit of a typical IR spectrometer (Figure 5).[^19^, ^74^] Even if their concentrations were 1000 times higher than the median, they would remain well below the threshold necessary to reliably link their contributions to spectral signals, especially given the between‐person biological variability of cell‐free blood.

Accordingly, when analyzing cell‐free blood, any observed changes in IR absorption cannot be attributed to variations in circulating DNA/RNA concentrations. It is important to note that DNA/RNA in tissues such as the liver, spleen, or heart can reach rather high concentrations that may shape IR spectra. Therefore, the conclusions drawn from this analysis should not be generalized to other biological specimens.

### Chemical Modifications of Molecules

**Figure 6:**
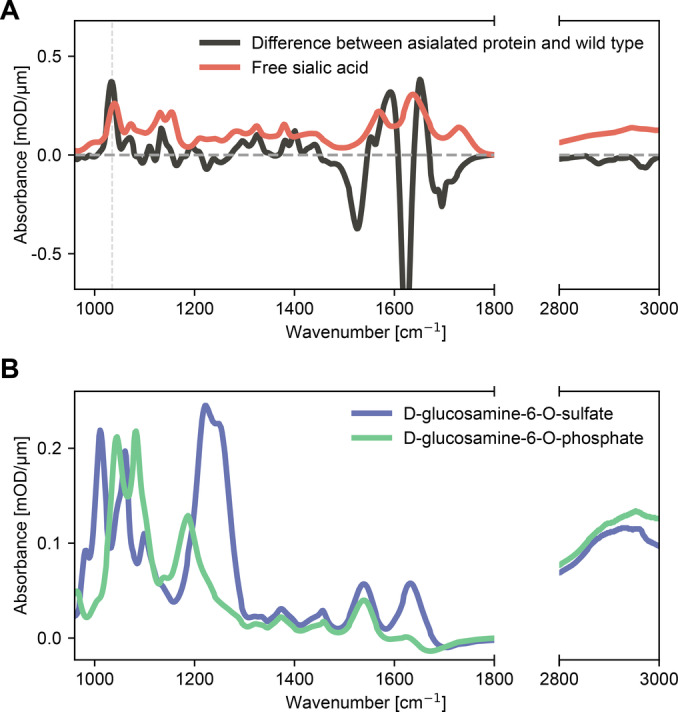
Impact of chemical modifications on IR spectra. (**A**) Absorption spectrum of free sialic acid (N‐acetylneuraminic acid, red) compared to the absorption differences obtained by subtracting the IR spectrum of asialofetuin (the desialylated form of fetuin) from that of fetuin (black). (**B**) Absorption spectra of D‐glucosamine‐6‐O‐sulphate (blue) and D‐glucosamine‐6‐O‐phosphate (green).

Some attempts to interpret changes between two groups of vibrational signatures rely on the spectra of cell‐free blood components recorded in isolation from their environment or on tables of characteristic vibrational modes.[^56^, ^57^, ^59^, ^60^, ^67^, ^75^] This approach overlooks the interactions between various blood components, such as extracellular molecular transport by effective diffusion or assembly into higher‐order complexes that modulate and regulate their dispersal (e.g., via proteins or encapsulation of molecules in extracellular vesicles).[^42^, ^76^] This very study also suffers from this limitation to some degree: The formation of non‐covalent macro‐molecular complexes may lead to shifts in vibrational frequencies that are overlooked when merely aggregating the contributions of individual components. However, a much larger change in the IR spectroscopic signatures of molecules, especially small ones, can be caused by the formation of additional *covalent* bonds. Here, we consider two examples of the many modifications that molecules undergo in their life cycle (Figure 6).

Protein glycosylation is a complex post‐translational modification that is sensitive to changes in the health status of individuals.^[77]^ For instance, altered sialylation has been reported in multiple cancers and liver diseases.^[77]^ The IR absorption spectrum of N‐Acetylneuraminic acid – the most common sialic acid – is depicted in Figure 6A. The sharp peak at 1730 cm^–1^ has been previously assigned to the −COOH vibration of sialic acid and used to monitor the degree of protein sialylation.[^78^, ^79^, ^80^] We then compared the IR absorption spectrum of bovine fetuin with its desialylated form, asialofetuin. The difference between their spectra clearly lacks the sharp peak at 1730 cm^−1^ that is apparent in free sialic acid (Figure 6A). Instead, the peak at 1035 cm^−1^ may be used to characterize the presence of sialic acid. In a complex mixture, however, this peak would at least partially be masked by the variability of other glycans and thus not detected.

The region of the IR spectrum that is often referred to as “carbohydrate” is also sometimes labeled as “fingerprint” – because it is sensitive to the overall changes in the molecular structure. Although the absorption pattern there can be used to confirm the presence of a specific molecule,^[1]^ the change in this pattern due to a chemical modification of the molecule is challenging to predict without quantum chemical calculations. To illustrate this, we compared the IR absorption spectra of substituted glycans: D‐glucosamine‐6O‐sulphate and D‐glucosamine‐6O‐phosphate (Figure 6B). The difference in their functional groups causes a shift in the characteristic asymmetric stretching band from 1186 cm^−1^ to 1222 cm^−1^, as expected. However, in addition to that, there was a significant shift of all the bands below 1200 cm^−1^. It is crucial to note that all these changes were caused by one chemical modification, with no changes in the carbohydrate content.

The presented examples serve as a reminder that vibrational spectra of molecular structures, especially small ones, are highly sensitive to their surroundings and chemical modifications. This is especially true for the “fingerprint” region of the IR spectrum, but not limited to it. It is also important to acknowledge that post‐translational modifications, such as sulphation, phosphorylation, methylation, glycosylation, etc., contribute not only their specific IR signatures directly, but might also affect other spectral regions by changing the 3D structure of the whole macromolecule.

### Effects of Measurement Preprocessing

To further compound the issue, molecular interpretation in spectral datasets is often performed without considering the preprocessing steps performed prior to data analysis. Common preprocessing steps include vector/peak normalization, baseline correction, offset correction, and smoothing – as comprehensively described in prior work.[^28^, ^81^, ^82^] While such data manipulations can help reduce noise and make datasets more comparable, some preprocessing steps can lead to artifacts in the analysis, including correlations in the noise structures of the data.^[28]^ These artifacts can result in false peaks that range from being very apparent in the data to being subtle and hard to detect. Here, we considered vector normalization (L2) as an example of a widely applied method to improve measurement reproducibility[^13^, ^28^, ^81^, ^83^] and demonstrate how it can mislead peak interpretations.

**Figure 7:**
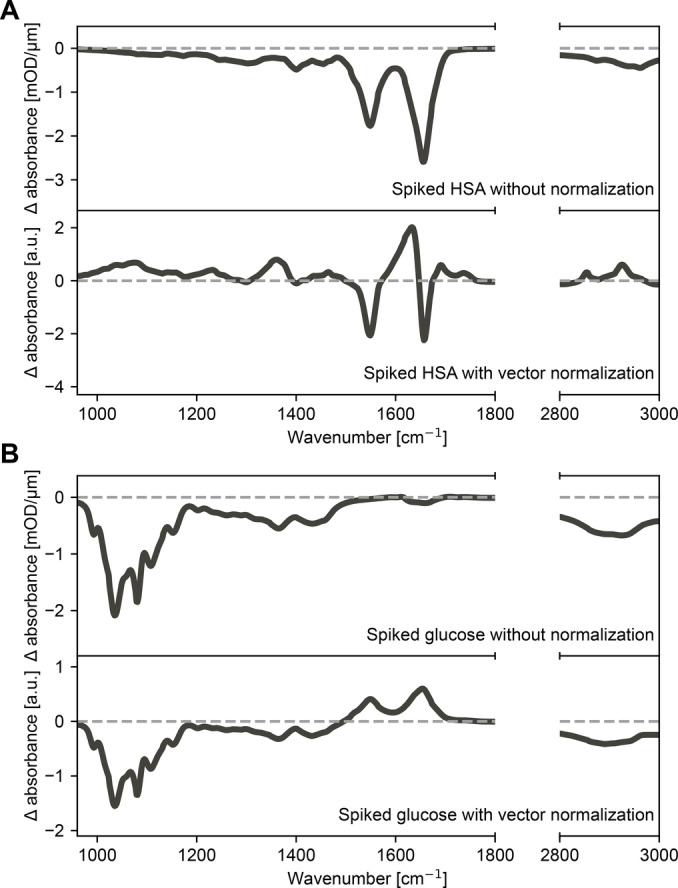
Effect of vector normalization on spectral interpretations. (**A**) Human serum albumin (HSA) was spiked to a typical blood serum spectrum. The curves depict the difference between the unspiked and spiked spectra, with and without vector normalization. (**B**) Same investigation, but spiking the spectrum of glucose instead of HSA.

Consider a case‐control scenario in which only the concentration of one molecule changes between two groups of samples. To model such a situation computationally, we spiked (added) the spectrum of HSA to a typical spectrum of blood serum. We then calculated the difference between the unspiked and spiked spectra (Figure 7A). We performed this analysis both with and without the normalization step, applied prior to subtraction. Very evidently, the resulting signal becomes hard to interpret when normalization was used, and associating this signal to the change in the HSA concentration would be nearly impossible. Although the negative absorbance near the amide‐I and ‐II regions was somewhat preserved in the normalized depiction, artifacts of positive “absorbance” were introduced across the remaining regions of the spectrum.

We then repeated the above analysis, but computationally spiking in the glucose spectrum instead (Figure 7B). The shape of the signal in the region between 960–1200 cm^−1^ was mostly preserved between the normalized and non‐normalized depictions. However, artifacts of positive absorbance now appeared near the amide‐I and ‐II regions – despite the absence of any significant absorbance in the spectrum of glucose in this region. Such an artifact could easily mislead one to think that there was a difference in protein content between the two groups of spectra. In reality, only the glucose concentration was altered.

Generally, applying measurement preprocessing steps may be necessary, particularly also to Raman spectroscopy.^[84]^ Therefore, we do not suggest to eliminate these steps. Rather, in conjunction with Morais et al.,^[28]^ we advocate for their cautious use, considering their potential impact on the data analysis. In some instances, this impact could make peak interpretations simply unfeasible.

### Univariate and Multivariate Spectral Analysis

**Figure 8:**
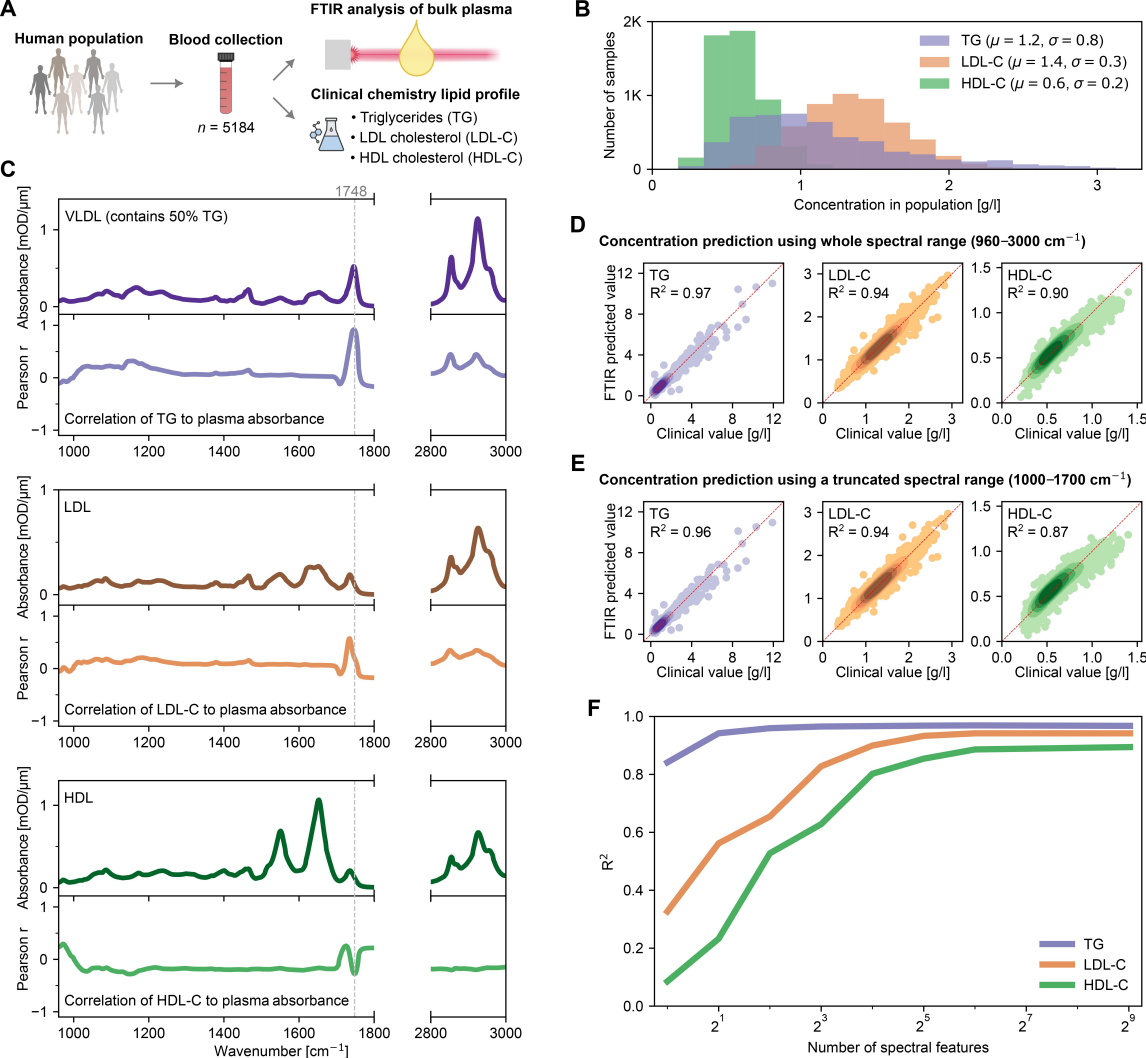
Unraveling the informational content of complex spectra with univariate and multivariate statistical analysis. (**A**) Blood samples were collected from a large, population‐based cohort for FTIR spectroscopy and clinical laboratory analysis to determine lipid analyte concentrations. (**B**) Distributions of lipid analyte concentrations in the population. (**C**) Absorbance spectra of single lipid particles (darker colors, upper panels) and the correlation between plasma absorption to the concentrations of the three lipid analytes (brighter colors, lower panels). (**D**) Multivariate regression models were trained to quantify the concentrations of each lipid analyte given an input spectrum of plasma. Each point represents the model's prediction for a plasma sample against the clinical measurement. A 10‐fold cross‐validation was applied. Only the predictions of the test sets of the cross‐validation splits are depicted. (**E**) Same multivariate regression analysis, but using a truncated spectral range as input to the model here. (**F**) Dependency between the number of spectral features given to the regression model and the quantification efficiency of each lipid analyte. A forward feature selection algorithm was applied to determine the optimal spectral features at each point. All correlation and regression analyses were applied on non‐normalized spectra.

The task of analyzing complex vibrational spectra necessitates robust statistical methods that can effectively uncover subtle yet critical spectral information often masked by overlapping signals. For instance, the power of multivariate statistical methods has long been recognized in spectral data analysis, including in foundational work of FTIR spectroscopy.^[85]^ Several studies offer constructive tutorials and guidelines on selecting appropriate data analysis methods for spectral data.[^1^, ^7^, ^24^, ^26^, ^27^, ^28^, ^86^] Here, we do not go through an extensive description of the technicalities of data analysis methods. Instead, we focus on their capacity to capture information on molecular substances from spectral datasets. First, we demonstrate how simple univariate analysis can be insightful, and then we point out its limitations, which necessitate the application of more advanced multivariate methods (Figure 8).

As an example, we revisited one of our previous studies.^[14]^ There, blood plasma from a large population‐based cohort was profiled with FTIR spectroscopy (Figure 8A). Laboratory clinical chemistry analyses were performed on the same blood samples to quantify the concentrations of triglycerides (TG), LDL cholesterol (LDL−C), and HDL cholesterol (HDL−C) (Figure 8A,B). This allowed us to correlate the information content of the plasma IR spectra with the clinically determined concentrations of these lipid‐centric analytes.

First, we calculated the Pearson correlation coefficient (*r*) between the concentrations of the three analytes and the IR absorbance observed in bulk plasma at each univariate spectral feature (Figure 8C). As a reference, we compared this correlation spectrum with that of the individual substance spectrum relevant to each analyte. Given that TG constitutes 50 % of VLDL dry mass,^[43]^ we compared the correlation of TG with that of the VLDL spectrum.

For TG and LDL−C, we found a high level of agreement between the univariate correlation spectrum and that of the individual lipid particles (Figure 8C). In particular, the narrow region between 1745–1750 cm^−1^ revealed a strong correlation between bulk plasma absorbance and the concentrations of the two substances. Specifically for TG, the peak at 1748 cm^–1^ aligned with that of the VLDL reference spectrum and at a very high correlation (r=0.92 ). This suggested that spectral signals relating to TG at this peak were largely not masked by the presence of any other substances. Surprisingly, however, the region of 2800–3000 cm^−1^ revealed a lower correlation level compared to the aforementioned region of 1745–1750 cm^−1^ for both TG and LDL−C. Given that the spectra of the pure substances have peaks with the strongest intensity between 2800–3000 cm^−1^, this suggested that the contributions of VLDL and LDL were overshadowed by other substances that also contribute spectral signals within that region, e.g., proteins, in line with Figure 1B,G.

In contrast, HDL−C revealed relatively weak correlations across the whole spectral range (Figure 8C). At the amide bands, for instance, no significant correlation strength was observed. This suggested that the plethora of other proteins in cell‐free blood masked the signals resulting from HDL. Similarly, in the regions between 1745–1750 cm^−1^ and 2800–3000 cm^−1^, no strong correlation was observed. This suggested that the signal of HDL−C was, again, overshadowed by the presence of other substances – namely, TG and LDL−C – which together have a much higher concentration than HDL−C (Figure 8B). Altogether, this analysis demonstrated that the HDL−C concentration had no single distinctive spectral feature that could be reliably linked to its concentration, unlike TG and LDL−C.

Compared to the prior analyses that relied on a relatively simple univariate approach, multivariate analysis is capable of considering the interactions among features. Such an aspect provides a more granular view of the information contained within the complex spectra as subtle dependencies between the absorption peaks can be captured – ones that would be otherwise obscured in a univariate context. To put this into practice, we trained a ridge regression model to quantify the clinically determined concentrations of the three lipid analytes given an input plasma spectrum (Figure 8D). When all the features in the spectral range were considered, a very high quantification efficacy was observed for all three analytes, with R^2^ values ranging from 0.90 to 0.97. Notably, even HDL−C was well quantified despite its lack of single distinctive features. This analysis highlights the power of multivariate algorithms to extract comprehensive insight from the information‐rich IR spectra.

It is also crucial to recognize that the information across several spectral features is highly co‐dependent. As previously discussed, individual substances can have many peaks across a wide range of frequencies. To illustrate the magnitude of this point, we performed the above regression analysis again, but paradoxically, using only the spectral range between 1000–1700 cm^−1^ – i.e., the range that had minimal univariate correlation to the lipid analytes. Surprisingly, the quantification efficacy for TG, LDL−C, and HDL−C remained nearly unchanged (Figure 8E). This further demonstrates the magnitude of the multi‐colinearity present within the spectral features and highlights that linking biological substances to specific spectral bands is highly questionable – especially when multivariate analysis methods are employed.

As demonstrated above, considering a large number of spectral features provides a more accurate overview of the breadth of molecular information in the IR spectra. To further examine this, we quantitatively determined the number of spectral features that were required to effectively quantify the concentrations of the lipid analytes. We examined the quantification efficacy for each studied analyte, considering a varying amount of spectral features given to the regression model (Figure 8F). These features were iteratively selected by a forward selection algorithm to determine the most optimal input features at each point. For TG, utilizing only one distinctive feature (1748 cm^−1^) had already resulted in a high quantification efficacy. Including just one more feature resulted in a nearly saturated quantification efficacy, revealing that the signal of TG can be adequately isolated using only two spectral features. Comparatively, for LDL−C and HDL−C, utilizing one or two spectral features proved to be insufficient. Specifically for HDL−C, nearly 64 different spectral features were required to reach the full potential of correlating its signal to the plasma spectra. Given that most substances in blood likely do not have such a distinctive feature as TG does, utilizing a low number of features or, e.g., considering spectral ratios, is therefore unlikely to provide a comprehensive view of the spectral informational content.

To summarize, when relating an IR absorption spectrum of a bulk mixture to certain components of interest:

1. univariate analysis (or use of a limited selection of peaks) is only appropriate when the signal of the substance is very strong and not masked by other compounds;
2. multivariate analysis may be capable of capturing information about a substance that is heavily overshadowed by the contributions of other compounds;
3. spectral features are highly co‐dependent, which allows for analyzing the signature of substances in spectral regions where they do not have prominent peaks; and
4. to harness the power of multivariate analysis, a substantial amount of spectral features are needed to discern complex feature inter‐dependencies (beyond spectral ratios).

### Navigating the Pitfalls of Spectral Interpretations

The question of how to most effectively analyze vibrational spectra of complex biological samples has no straightforward answer. Each analytical task is unique, targets different biological phenomena, is specific to different medical scenarios, and faces different requirements and challenges. Generally, we as a community would like to have a realistic overview of the molecules and their combinations we are profiling when analyzing complex biological matrices. Equally important is raising awareness about what we cannot possibly observe as it is beyond the technical limitations of the current technology and analytical setting. Furthermore, we would like to develop robust analytical workflows that accurately model alterations in a studied system (e.g., characteristics of the human phenotype). Below, we discuss how to approach these tasks.

**Figure 9:**
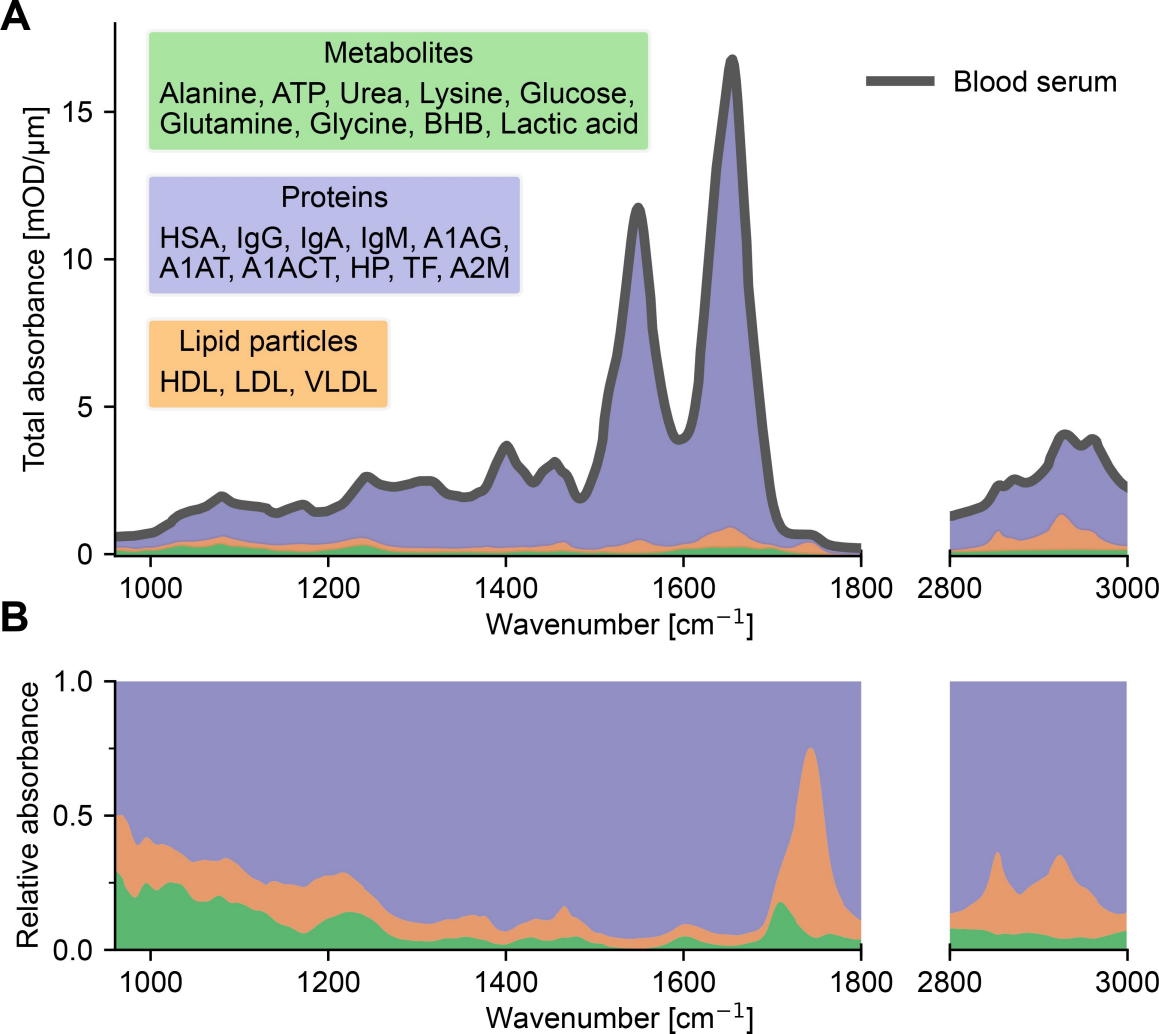
Composite makeup of a typical infrared spectrum of venous blood serum. (**A**) Stacked representation of the primary molecular classes found in cell‐free blood. Individual component spectra of each substance were scaled according to their typical concentrations in healthy adults (Supporting Information Table S1) and combined to estimate the cumulative spectral contributions. Molecular classes were stacked in sequence, with metabolites at the base, followed by lipid particles, and proteins on top. Black curve represents the contribution of their total sum, approximating the spectrum of blood serum. Supplementary Figure S3 compares the approximated spectrum with a true serum spectrum. (**B**) Relative absorption contribution of each molecular class, illustrating the proportions of total absorption across the spectral range. *ATP ‐ Adenosine triphosphate; BHB ‐ Beta‐hydroxybutyric acid; HSA ‐ Human serum albumin; IgG ‐ Immunoglobulin G; IgA ‐ Immunoglobulin A; IgM ‐ Immunoglobulin M; A1AG ‐ Alpha‐1‐acid glycoprotein; A1AT ‐ Alpha‐1‐antitrypsin; A1ACT ‐ Alpha‐1‐antichymotrypsin; HP ‐ Haptoglobin; TF ‐ Transferrin; A2 M ‐ Alpha‐2‐macroglobulin*.

First, to produce a more realistic view of the composition of the IR spectrum from cell‐free blood, we decomposed it into the contributions of its primary molecular classes: metabolites, proteins, and lipid particles (Figure 9A). It is evident that proteins dominate the absolute absorption signals across the spectral range, considering that lipid particles also contain a proportion of proteins. When examining the relative contributions of each molecular class, their influence on absorption signals significantly varies across the spectral range (Figure 9B). These representations should not imply that one molecular class provides more information over another. For example, glucose, a metabolite, can be reliably determined in blood plasma, serum, and whole blood using IR spectroscopy as previously mentioned.[^14^, ^52^, ^53^, ^54^, ^55^] Rather, these representations further underscore the notion that the IR spectrum should not be overly simplified into discrete regions exclusively linked to specific molecular classes. Each substance and each molecular class contributes to the composite nature of the overall IR spectrum, which offers a holistic view of the molecular landscape within the sample.

When confronted with changes in the IR absorption spectrum of a biological sample, researchers may be tempted to seek simple, intuitive explanations. However, as demonstrated in our examples, such approaches should be avoided unless robustly validated. Machine learning and multivariate statistical methods are instrumental in scenarios where the molecular components are numerous and the relationships between them are complex. Nevertheless, it is crucial that these statistical models are trained on reproducible, high‐quality, well‐characterized datasets and that the results are reasonable within the biological context of the study. This typically requires large datasets that accurately reflect demographic distributions, include appropriate control reference samples, and involve diverse pathophysiological strata (e.g., comorbidities or stages of a disease). Furthermore, biological systems are inherently dynamic, and thus, statistical models should be regularly updated with new data. Adapting and learning from new inputs will help improve the statistical models’ accuracy and relevance. This is especially important in clinical, medical settings, where training data must mirror realistic data variations caused by inherent biological variabilities (e.g., lifestyle changes and aging), sample collection variations, differences in sample storage regimes, measurement device maintenance, and other technical variations typically encountered in practice.^[87]^ Furthermore, independent external validation of developed models on samples from different clinical studies is essential to more robustly determine whether results are reproducible.

If molecular interpretations of changes in the IR absorption spectrum are necessary, we suggest involving complementary analytical techniques. Techniques such as mass spectrometry, nuclear magnetic resonance (NMR)‐based omics, and clinical chemistry provide orthogonal information that aids in this process.[^14^, ^29^, ^88^, ^89^] Another fruitful avenue is to reduce the molecular complexity via chemical fractionation – e.g., liquid chromatography,^[30]^ protein precipitation^[29]^ or ultrafiltration.[^10^, ^31^, ^32^, ^33^] IR measurements can then be performed on the individual fractions, which are less complex in terms of their molecular makeup and might be easier to interpret. These strategies offer independent confirmation of molecular identities and concentrations, enabling further verification of the findings. Furthermore, the results should be compared to other biomarker research efforts, and the molecular‐specific mechanistic and pathway information that the medical community sometimes requires should be provided. Once the molecular mechanisms are provided and validated, the IR approach can be used in its high‐throughput mode – without the involvement of orthogonal measurement approaches.

Generally, the conceptional suggestions presented here can be broadly applied to IR spectroscopic examinations of other complex biological systems beyond blood plasma. Extensions to model paradigms such as yeast, bacterial, algal, and mammalian cells, as well as animal, human, and plant tissues can be envisioned. However, it is important to consider that the IR spectral signatures of different model paradigms can significantly vary due to their distinct molecular makeup (e.g., the content of proteins vs. carbohydrates is different between animal and plant models^[90]^) and there may be instances where the assignment of spectral regions to specific molecular entities is feasible. For instance, certain wavenumbers in cellular spectra can be qualitatively linked to nucleic acid content,^[91]^ or sialic acid content may be identified at specific wavenumbers in spectra of extracellular polymeric substances.^[79]^ Nevertheless, spectral molecular assignments must be performed cautiously and, ideally, supplemented with additional experimentation, as previously discussed.

Transparency and the critical evaluation of the reported results are also essential aspects. Documentation of all analytical steps and assumptions for interpretations should be clearly communicated in peer‐reviewed works. For instance, it is crucial to detail the data preprocessing steps and their impact on the observed spectral signals. The spectroscopic technique's dynamic range and sensitivity limits must always be considered and recognized, especially when dealing with low‐abundant substances. Another rarely discussed aspect revolves around the sample collection methodology (e.g., if a variety of sample collection tubes were used within the same study). For instance, blood plasma collection tubes, as opposed to serum, use ethylenediaminetetraacetic acid (EDTA) or other anticoagulants within the blood draw tubes. EDTA typically has a high enough concentration that it contributes to absorption signals of bulk plasma spectra to a significant extent (Supplementary Figure S4). Changes in the blood draw and sample processing workflows or the use of samples from different clinical studies and clinical centers could therefore lead to noticeable variations in the signals when studying certain biological phenomena, potentially confounding the results.

Finally, the value of multidisciplinary research must be recognized. A purely chemical, spectroscopic perspective is no longer sufficient on its own. It must be complemented by expertise in biological/medical sciences, computational analyses, and physical interpretations. Encouraging collaboration across these disciplines integrates knowledge from different perspectives. Collaborative efforts can drive innovative solutions to the challenges of interpreting complex spectra and lead to the development of new analysis techniques.

## Conclusion

In this perspective, we highlighted the critical yet often overlooked intricacies of analyzing vibrational spectral data, particularly IR spectroscopy of cell‐free blood. The method's capacity to provide molecular fingerprints is unmatched in its simplicity, speed, and molecular breadth – making it a prime candidate for inclusion in high‐throughput diagnostic workflows. To bring the technology further, and to mitigate it to practical use in possible medical diagnostics, its limitations, however, must always be recognized.

Our illustrative case studies utilized thousands of blood‐based spectra in addition to spectra of predominant proteins, major lipid particles, and key metabolites. These single‐component spectra together make up the primary components of cell‐free blood. Yet, we acknowledge that this is not an exhaustive list of all blood constituents and that our approach did not consider inter‐molecular interactions.^[92]^ Nonetheless, by expanding the scope of substances analyzed and considering the interactions between these molecular components, isolating the signal of specific molecules in complex mixtures becomes even more challenging, as our analysis demonstrates.

Moving forward, we advocate caution when performing IR spectral interpretations to avoid speculative conclusions that could undermine the applicability as well as acceptability of research, particularly in clinical settings. Instead, the high‐throughput fingerprinting nature of the approach should be accepted. By embracing both its capabilities and limitations, we can more effectively establish robust spectroscopic analytical workflows.

## Conflict of Interests

The authors declare no conflict of interest.

1

## Biographical Information

*Tarek Eissa began his studies in Computer Science at the American University in Cairo before moving to the Technical University of Munich, where he earned his master's in Data Engineering and Analytics. He is currently completing his PhD with the Broadband Infrared Diagnostics (BIRD) team, jointly between the Chairs of Laser Physics, Ludwig Maximilians University in Munich, and Bioinformatics, Technical University of Munich. Driven by his interest in data science, Tarek's research centers on analyzing blood‐based infrared spectra for clinical diagnostics*.

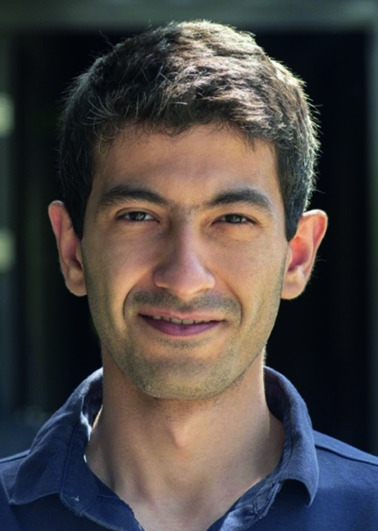

## Biographical Information

*Liudmila Voronina studied at the Moscow Institute of Physics and Technology, before moving to the Swiss Federal Institute of Technology Lausanne, where she obtained her PhD in Physical Chemistry. In 2017, she joined the Broadband Infrared Diagnostics group at the Chair of Laser Physics, Ludwig Maximilians University in Munich as a Postdoc.Mobility fellow of the Swiss National Science Foundation. Liudmila has since become a senior scientist, expanding her research interests from spectroscopy of individual biomolecules to applications of liquid biopsies*.

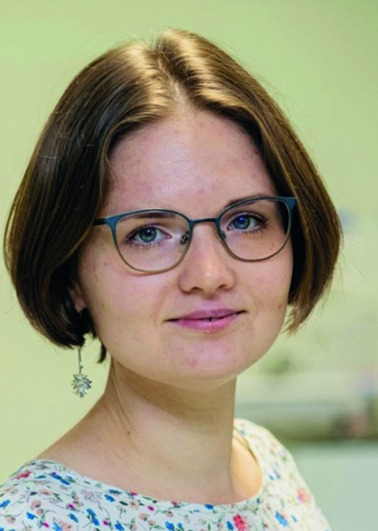

## Biographical Information

*Marinus Huber studied Physics at the Friedrich Schiller University of Jena and the Ludwig Maximilians University in Munich, completing his master's thesis at Harvard University. For his PhD at Ludwig Maximilians University, he developed new laser‐based infrared spectroscopy methods for medical diagnostics. He continued this work as a postdoc at the Max Planck Institute of Quantum Optics, Friedrich Schiller University, and the University of Kaiserslautern‐Landau, focusing on innovative applications of infrared spectroscopy in analyzing liquids and cells*.

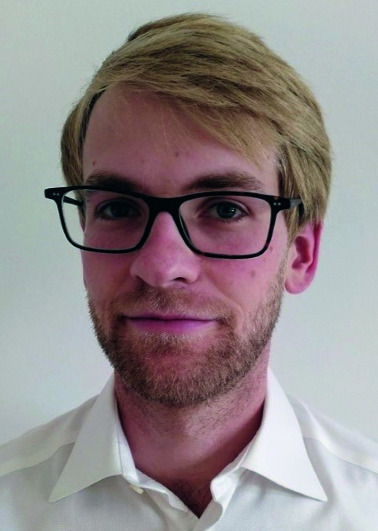

## Biographical Information

*Frank Fleischmann studied Biology at the Technical University of Munich, and earned his PhD in Phytopathology from the Technical University of Munich. Following his postdoctoral research in forest pathology at the same university, he worked with the genetics service provider IMGM Laboratories in Martinsried. In 2018, Frank joined the Broadband Infrared Diagnostics (BIRD) team at the Chair of Laser Physics, Ludwig Maximilians University in Munich, where his research focuses on establishing and employing infrared molecular spectroscopy to fingerprint complex human samples*.

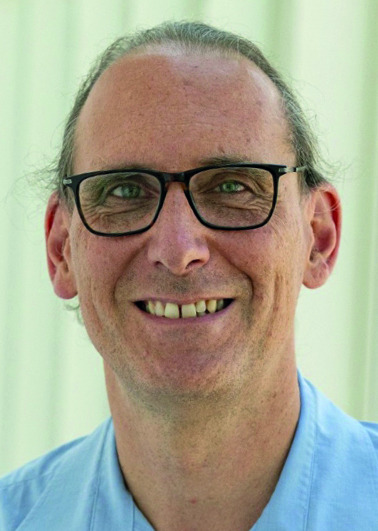

## Biographical Information

*Mihaela Žigman studied Molecular Biology at the University of Ljubljana and earned her PhD from the University of Vienna. During postdoctoral research at the Institute of Molecular Biotechnology of the Austrian Academy of Sciences in Vienna, and at the Fred Hutchinson Cancer Research Center in Seattle, she investigated molecular mechanisms controlling cell division. Following her research at the Ruprecht Karls University in Heidelberg, she joined the Ludwig Maximilians University in Munich in 2015. Mihaela established the Broadband Infrared Diagnostics (BIRD) research, leveraging infrared molecular spectroscopy to analyze and map human health*.

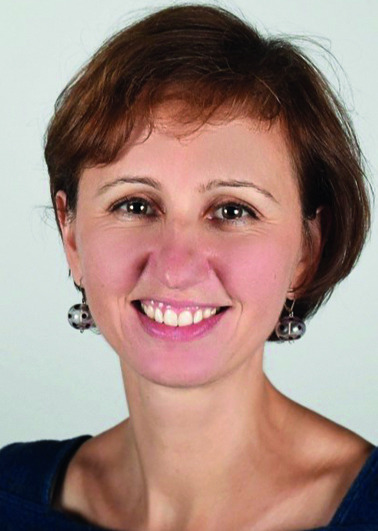

## Supporting information

As a service to our authors and readers, this journal provides supporting information supplied by the authors. Such materials are peer reviewed and may be re‐organized for online delivery, but are not copy‐edited or typeset. Technical support issues arising from supporting information (other than missing files) should be addressed to the authors.

Supporting Information

## Data Availability

The data that support the findings of this study are available on request from the corresponding author. The data are not publicly available due to privacy or ethical restrictions.
