# Supplementary material for: The Perils of Molecular Interpretations from Vibrational Spectra of Complex Samples
Source: Angew Chem Int Ed Engl. 2024 Nov 7;63(50):e202411596. doi: 10.1002/anie.202411596 (PMC11610679; doi:10.1002/anie.202411596)
Supplement: Supplementary file 1 — Supporting Information [file ANIE-63-e202411596-s001.pdf]

Angewandte Chemie
Eine Zeitschrift der Gesellschaft Deutscher Chemiker

Supporting Information

The Perils of Molecular Interpretations from Vibrational Spectra of Complex Samples

*T. Eissa, L. Voronina, M. Huber, F. Fleischmann, M. Žigman**

Material and Methods

Blood sample collection and processing

All blood samples utilized in this work were considered in our previous work [1–3], where details on the clinical studies, sample collection, and sample processing were provided.

Details relating to lung cancer and proteomic analysis (Fig. 4) were provided in Voronina et al. [2] Lung cancer detection was a part of a multi-institutional study on lung, breast, bladder, and prostate cancers with corresponding benign pathologies in the same organs as well as non-symptomatic subjects [1]. Participants provided written informed consent for the study under research study protocol #17-141 and broad consent under research study protocol #17-182, both of which were approved by the Ethics Committee of the Ludwig-Maximillian-University (LMU) of Munich. The study complies with all relevant ethical regulations and was conducted according to Good Clinical Practice (ICH-GCP) and the principles of the Declaration of Helsinki. The clinical trial is registered (ID DRKS00013217) at the German Clinical Trails Register (DRKS).

Details relating to the lipid parameter analysis (Fig. 8) were provided in Eissa et al. [3] Blood samples were collected as a part of the Cooperative Health Research in the Region of Augsburg (KORA) study [4] – which comprised of an age- and sex-stratified sample of participants randomly drawn from the resident registration offices within Sourther Germany. The KORA study methods were approved by the ethics committee of the Bavarian Chamber of Physicians, Munich (EC No. 06068).

Chemicals, reagents, and measurements of infrared absorption spectra

Proteins and lipid particles of the highest available purity were purchased from Sigma Aldrich GmbH (Taufkirchen, Germany). They were diluted in deionized water to concentrations of 1-5 mg/mL, and their IR absorption spectra were measured. If the presence of additional salts was detected, the buffer was exchanged to deionized water using Amicon Ultra centrifugal filters with 10 kDa cutoff, purchased from Sigma Aldrich GmbH (Taufkirchen, Germany). The concentration of the proteins in the resulting solution was then measured using a Nanodrop One UV-Vis spectrophotometer (Thermo Fischer Scientific GmbH, Dreieich, Germany), and the UV absorption of the solution before buffer exchange was used for device calibration.

To assess how the pH of the solution and the resulting protein protonation state affected the IR spectra of the proteins, we recorded the absorption spectra of several model proteins in PBS buffer (pH 7.4). We then subtracted the IR spectrum of the buffer from that of the protein in a buffer in order to obtain the IR signature of a pure protein in a controlled protonation state. When comparing these spectra with the ones recorded in pure water, we did not observe any significant differences in the ratios of the primary absorption bands, their shapes, or intensities. Based on experiences across a variety of our experimentation, we consider that the details of the IR spectra of the proteins related to the pH would not affect any of our conclusions.

The water-soluble metabolites were purchased from Sigma Aldrich GmbH (Taufkirchen, Germany), diluted in deionized water, and their IR absorption was measured. The same procedure was followed for glycans, purchased from Dextra Laboratories (Reading, UK).

Pure DNA from calf thyme and RNA from bakers yeast were purchased from Sigma Aldrich GmbH (Taufkirchen, Germany) and dissolved in deionized water. Their concentration and purity (A260/280 ratio >1.9) were assessed using a Nanodrop One UV-Vis spectrophotometer with the built-in methods for double-stranded DNA and single-stranded RNA. Based on NanoDrop results, the concentrations were adjusted to 1 mg/mL for the FTIR measurement. The obtained spectra were scaled down to the concentrations shown in Fig. 5, as those are below the detection limit.

Measurements of liquid biofluids and single components were all performed in a hydrated, fluid state using an automated FTIR device MIRA-Analyzer (Clade GmbH, Esslingen, Germany) with a flow-through transmission cuvette (CaF_2 with 8 µm path length). The spectra were acquired with a resolution of 4 cm^{-1} and an averaging time of 45 s. Prior to every sample, a water reference spectrum was measured to reconstruct the IR absorption spectra. The spectra were obtained in the range 930-3050 cm^{-1} and truncated to 960-3000 cm^{-1}.

Preprocessing of all absorption spectra was performed as follows. To account for the water substituted by the molecular constituents in the sample compared to the pure water reference, a water absorption spectrum was added to the sample spectrum with a coefficient optimized such that the first derivative of the signal in the region of 1850-2150 cm^{-1} was minimized, similar to our prior work [1–3,5]. Subsequently, the minimum of the absorption in this region was subtracted from the spectrum ("offset correction") [2]. Vector normalization was applied to the spectra in the analysis relevant to lung cancer (Fig. 4), as performed in the prior work [2]. Vector normalization (L2) was also applied in part of the analysis related to the normalization analysis (Fig. 7), as described in the figure legend.

Mathematical optimizations and statistical analyses

All mathematical and statistical analysis was performed in a Python environment (version 3.8.8) using custom scripts.

To reproduce a spectral signal given a set of substances (Figs. 3, 4, S2), the “minimize” function from the SciPy Python package (version 1.6.2) was used [6]. The objective function calculated the mean squared error between the target signal to be reproduced and the input set of spectra [Eq. 2].

For the regression analysis to quantify the concentrations of lipid analytes (Fig. 8), a linear regression algorithm with a ridge penalty was used. A cross-validation was performed on the available spectra to quantify the concentrations of the analytes which were determined from clinical chemistry analysis [3]. Mean R^2 values of the test predictions were reported, and all test set prediction errors were depicted (Fig. 8D,E). The Scikit-learn Python package (version 0.24.1) was used for its implementations of the ridge regression algorithm [7].

Table S1. Individual molecular substances considered in this work, consisting of ten proteins, three lipid particles, and nine metabolites, along with their typical concentrations in healthy adults.

Substance	Abbreviation	Concentration [g/l] [a]	Molecular class
Human serum albumin	HSA	42.65	Protein
Immunoglobulin G	IgG	9.55	Protein
Immunoglobulin A	IgA	3.36	Protein
Alpha-2-macroglobulin	A2M	3.20	Protein
Transferrin	TF	2.66	Protein
Haptoglobin	HP	1.74	Protein
Immunoglobulin M	IgM	1.71	Protein
Alpha-1-antitrypsin	A1AT	1.49	Protein
Alpha-1-acid glycoprotein	A1AG	1.03	Protein
Alpha-1-antichymotrypsin	A1ACT	0.55	Protein
Low-density lipoprotein	LDL	3.11	Lipid particle
Very low-density lipoprotein	VLDL	2.40	Lipid particle
High-density lipoprotein	HDL	2.00	Lipid particle
Adenosine triphosphate	ATP	1.52	Metabolite
D-Glucose	Glucose	0.90	Metabolite
Urea	Urea	0.24	Metabolite
Lactic acid	Lactic acid	0.09	Metabolite
Glutamine	Glutamine	0.07	Metabolite
Lysine	Lysine	0.05	Metabolite
Alanine	Alanine	0.04	Metabolite
Beta-hydroxybutyric acid	BHB	0.04	Metabolite
Glycine	Glycine	0.03	Metabolite

[a] Typical concentrations were estimated from existing literature for proteins [8–13] and metabolites [14]. Concentrations of lipid particles were estimated from the studied KORA population-based cohort (Fig. 8B) [3,4]. Specifically, HDL and LDL particle concentrations were estimated from HDL and LDL cholesterol levels – assuming that HDL and LDL particles are respectively made of 30% and 45% cholesterol and cholesteryl ester [15]. VLDL particle concentrations were estimated from triglyceride concentrations – assuming that triglycerides make up 50% of VLDL particles [15].

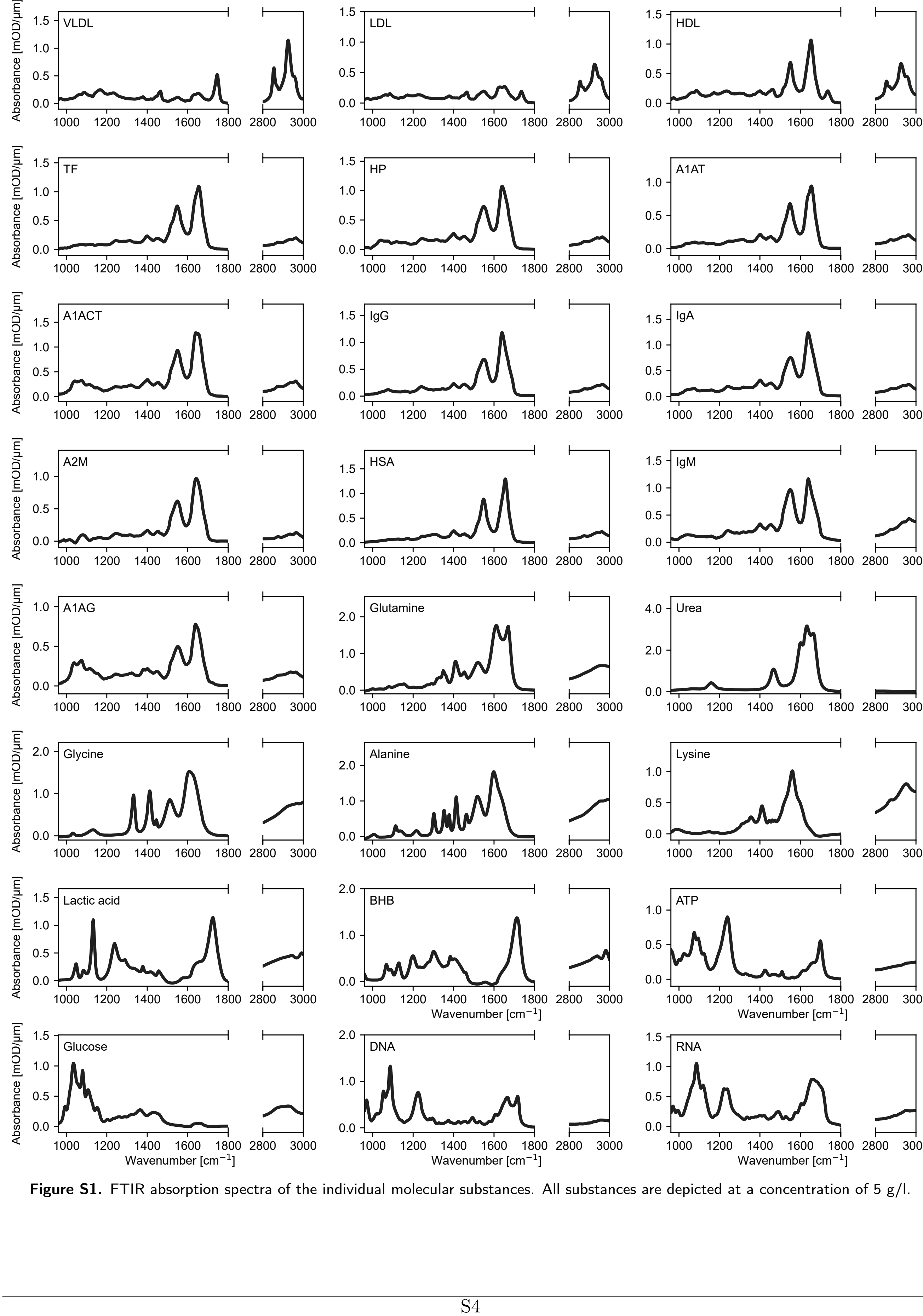

Figure S1. FTIR absorption spectra of the individual molecular substances. All substances are depicted at a concentration of 5 g/l.

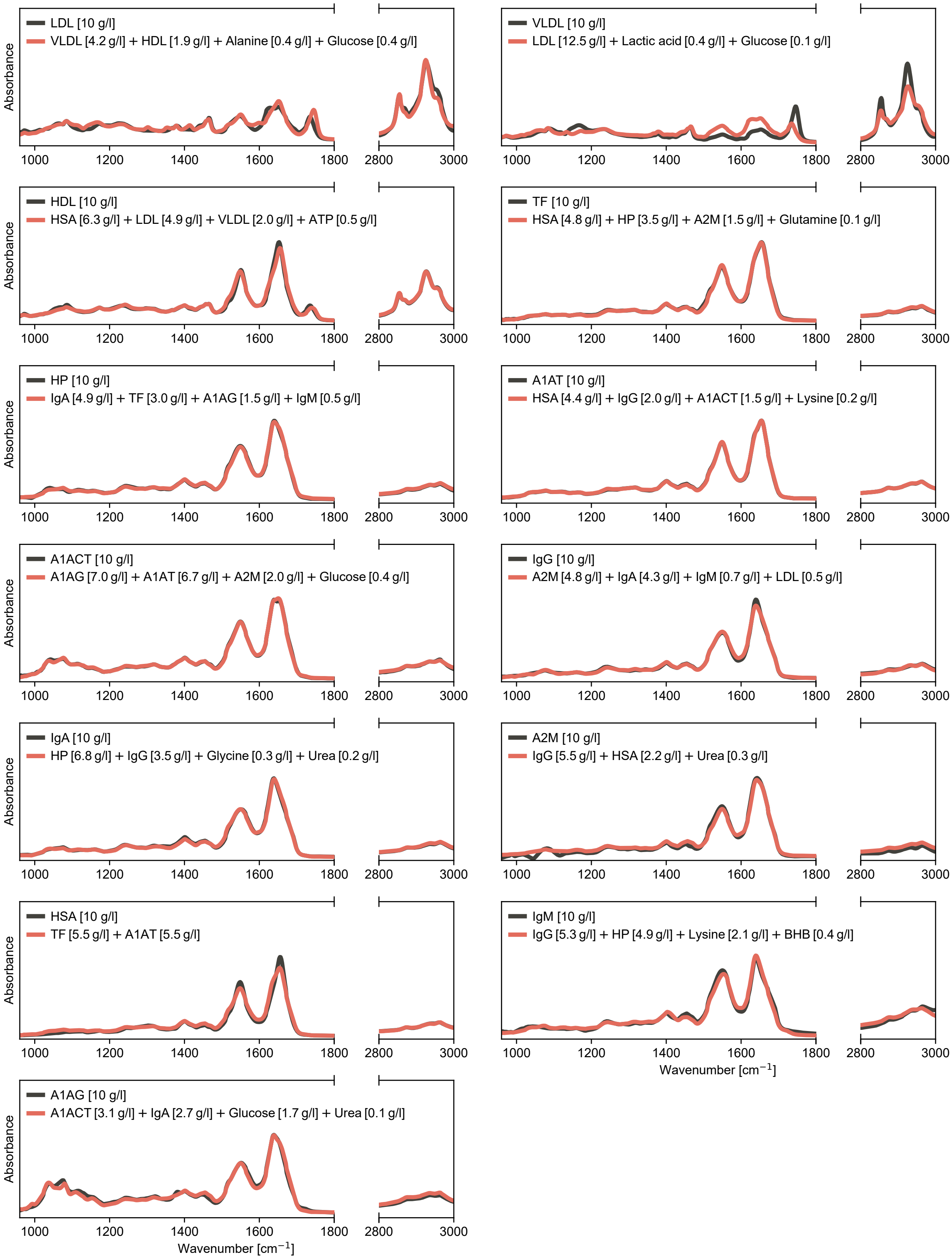

Figure S2. Reconstructing the spectrum of a single substance (black) from a linear combination of other substances (red) scaled to different concentrations. Related to Fig. 3.

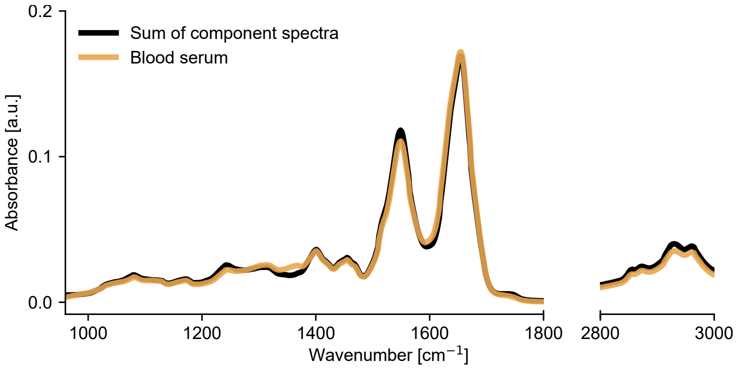

Figure S3. Comparing an experimental blood serum spectrum with a model constructed from individual component spectra. The modeled spectrum (black) was generated through the addition of individual component spectra of different metabolites, proteins, and lipid particles, each scaled to their typical concentrations in healthy adults (Table S1). The experimental spectrum (yellow) represents the mean spectrum of bulk serum from a nominally healthy adult cohort.

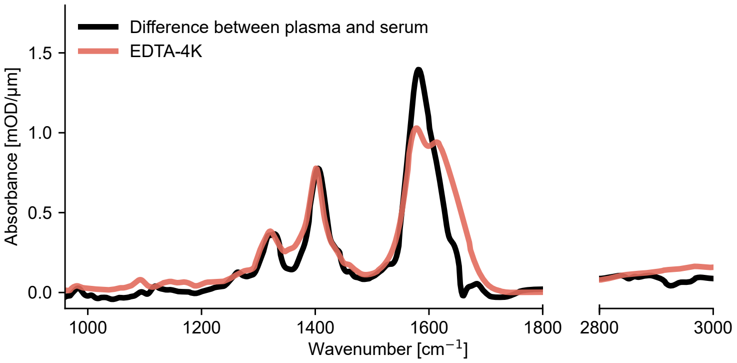

Figure S4. Difference between absorption spectra of blood plasma and serum. Both plasma and serum were processed from the same whole blood samples for different individuals. The black curve depicts the difference between plasma and serum spectra, processed from the same whole blood sample, and subsequently averaged across the different individuals. The red curve depicts the spectrum of EDTA-4K (Ethylenediaminetetraacetic acid tetrapotassium salt), the anticoagulant used for plasma collection. EDTA-4K was measured in a NaCl background and its spectrum was scaled to align the peak near 1400 cm^{-1}.

Supplementary References

[1] M. Huber, K. V. Kepesidis, L. Voronina, F. Fleischmann, E. Fill, J. Hermann, I. Koch, K. Milger-Kneidinger, T. Kolben, G. B. Schulz, F. Jokisch, J. Behr, N. Harbeck, M. Reiser, C. Stief, F. Krausz, M. Zigman, *eLife* **2021**, *10*.

[2] L. Voronina, C. Leonardo, J. B. Mueller-Reif, P. E. Geyer, M. Huber, M. Trubetskov, K. V. Kepesidis, J. Behr, M. Mann, F. Krausz, M. Žigman, *Angewandte Chemie International Edition* **2021**, *60*, 17060.

[3] T. Eissa, C. Leonardo, K. V. Kepesidis, F. Fleischmann, B. Linkohr, D. Meyer, V. Zoka, M. Huber, L. Voronina, L. Richter, A. Peters, M. Žigman, *Cell Reports Medicine* **2024**, *5*, 101625.

[4] R. Holle, M. Happich, H. Löwel, H. Wichmann, *Das Gesundheitswesen* **2005**, *67*, 19–25.

[5] M. Huber, K. V. Kepesidis, L. Voronina, M. Božić, M. Trubetskov, N. Harbeck, F. Krausz, M. Žigman, *Nature Communications* **2021**, *12*.

[6] P. Virtanen, R. Gommers, T. E. Oliphant, M. Haberland, T. Reddy, D. Cournapeau, E. Burovski, P. Peterson, W. Weckesser, J. Bright, S. J. van der Walt, M. Brett, J. Wilson, K. J. Millman, N. Mayorov, A. R. J. Nelson, E. Jones, R. Kern, E. Larson, C. J. Carey, İ. Polat, Y. Feng, E. W. Moore, J. VanderPlas, D. Laxalde, J. Perktold, R. Cimrman, I. Henriksen, E. A. Quintero, C. R. Harris, A. M. Archibald, A. H. Ribeiro, F. Pedregosa, P. van Mulbregt, SciPy 1.0 Contributors, *Nature Methods* **2020**, *17*, 261.

[7] F. Pedregosa, G. Varoquaux, A. Gramfort, V. Michel, B. Thirion, O. Grisel, M. Blondel, P. Prettenhofer, R. Weiss, V. Dubourg, J. Vanderplas, A. Passos, D. Cournapeau, M. Brucher, M. Perrot, E. Duchesnay, *Journal of Machine Learning Research* **2011**, *12*, 2825.

[8] R. F. Ritchie, G. E. Palomaki, L. M. Neveux, O. Navolotskaia, *Journal of Clinical Laboratory Analysis* **1998**, *12*, 371–377.

[9] R. F. Ritchie, G. E. Palomaki, L. M. Neveux, O. Navolotskaia, T. B. Ledue, W. Y. Craig, *Journal of Clinical Laboratory Analysis* **1999**, *13*, 273–279.

[10] R. F. Ritchie, G. E. Palomaki, L. M. Neveux, O. Navolotskaia, T. B. Ledue, W. Y. Craig, *Journal of Clinical Laboratory Analysis* **2000**, *14*, 284–292.

[11] A. Kratz, M. Ferraro, P. M. Sluss, K. B. Lewandrowski, *New England Journal of Medicine* **2004**, *351*, 1548–1563.

[12] J. Housley, *Journal of Clinical Pathology* **1968**, *21*, 27–31.

[13] F. Licastro, E. Masliah, S. Pedrini, L. J. Thal, *Dementia and Geriatric Cognitive Disorders* **2000**, *11*, 25–28.

[14] N. Psychogios, D. D. Hau, J. Peng, A. C. Guo, R. Mandal, S. Bouatra, I. Sinelnikov, R. Krishnamurthy, R. Eisner, B. Gautam, N. Young, J. Xia, C. Knox, E. Dong, P. Huang, Z. Hollander, T. L. Pedersen, S. R. Smith, F. Bamforth, R. Greiner, B. McManus, J. W. Newman, T. Goodfriend, D. S. Wishart, *PLoS ONE* **2011**, *6*, e16957.

[15] R. A. Davis, J. E. Vance, *Structure, assembly and secretion of lipoproteins*, page 473–493, Elsevier **1996**.
